# Medication errors related to transdermal opioid patches: lessons from a regional incident reporting system

**DOI:** 10.1186/2050-6511-15-31

**Published:** 2014-06-09

**Authors:** Henrik Lövborg, Mikael Holmlund, Staffan Hägg

**Affiliations:** 1 Department of Clinical Pharmacology and Department of Medical and Health Sciences, Linköping University, Linköping, Sweden; 2 Clinical Pharmacology, University Hospital, S-581 85 Linköping, Sweden

**Keywords:** Transdermal patch, Opioids, Fentanyl, Buprenorphine, Medication errors, Incident reporting system

## Abstract

**Objective:**

A few cases of adverse reactions linked to erroneous use of transdermal opioid patches have been reported in the literature. The aim of this study was to describe and characterize medication errors (MEs) associated with use of transdermal fentanyl and buprenorphine.

**Methods:**

All events concerning transdermal opioid patches reported between 2004 and 2011 to a regional incident reporting system and assessed as MEs were scrutinized and characterized. MEs were defined as “a failure in the treatment process that leads to, or has the potential to lead to, harm to the patient”.

**Results:**

In the study 151 MEs were identified. The three most common error types were wrong administration time 67 (44%), wrong dose 34 (23%), and omission of dose 20 (13%). Of all MEs, 118 (78%) occurred in the administration stage of the medication process. Harm was reported in 26 (17%) of the included cases, of which 2 (1%) were regarded as serious harm (nausea/vomiting and respiratory depression). Pain was the most common adverse reaction reported.

**Conclusions:**

Of the reported MEs related to transdermal fentanyl and buprenorphine, most occurred during administration. Improved routines to ascertain correct and timely administration and educational interventions to reduce MEs for these drugs are warranted.

## Background

The usage of transdermal patches which allows continuous and prolonged delivery of medications is increasing. Less frequent dosing, lower peak plasma drug concentration compared with other types of administration forms and avoidance of first-passage metabolism are some reported benefits suggesting that this administration form may have increased compliance, effectiveness and safety compared to oral administration [1,2]. In addition, patches offer the potential to deliver medications that would otherwise require injections. Moreover, future advances in the technology will probably increase the utilization of drug patches further.

Sporadic case reports indicate however specific problems related with this drug form, such as incorrect use of multiple patches [3,4], ingestion of used or unused patches [5] and skin reaction [6]. Since potent drugs linked to serious adverse reactions are administered through transdermal patches, medication errors may lead to serious adverse health consequences. The American Food and Drug Administration (FDA) states that opioids poses the highest risk of harm and death, among approved transdermal patches, because of their risk to cause respiratory depression [7] and has, along with other organizations, issued warnings regarding unsafe use of these patches [8,9]. There are also previous reports of serious and fatal cases due to erroneous administration of multiple patches of rivastigmine, an anticholinergic drug used to treat dementia [4,10]. Prevalence and characteristics of medications errors related to transdermal opioid patches has not been systematically compiled and presented in the scientific literature and such information is needed to develop effective preventive measures. This study was therefore undertaken to describe and characterize medication errors regarding transdermal opioid patches, containing fentanyl and buprenorphine, submitted to a regional incident reporting system.

## Method

### Data source

Reports on medication errors were identified in a web-based incident reporting system for the healthcare organization within the County Council of Östergötland, Southeastern Sweden. Permission to access the data was obtained from the patient safety department of the Couty Council. Ethical review was not required for this study, based on national legislation [11]. The database did not contain information that was directly or indirectly identifying individual subjects. The catchment area in this study comprised 431 075 persons (2011). All are encouraged to report all types of incidents and risks. Incident and risk were defined by the county council as “an unexpected event or observation that leads to or has the potential to lead to harm to a patient, relative or family member, employee, equipment, or organization”. In this study all events concerning transdermal opioid patches reported between 2004 and 2011 were scrutinized. The data included free text description of the incident, reporting clinic, and date of the incident.

### Cases

For incident reports to be included in this study they had to be classified as MEs according to the definition “a failure in the treatment process that leads to or has the potential to lead to harm to patients” [12], and concern transdermal opioid patches with fentanyl or buprenorphine as active ingredients (e.g. fentanyl in Durogesic and Matrifen or buprenorphine in Norspan). “Pain relief patch” is a commonly used term for these patches among healthcare employees and MEs described with this term have accordingly been included. To reliably identify reports when searching the database, the key terms used included the words *patch, fentanyl, buprenorphine, transdermal, Norspan, Durogesic, Matrifen* and a number of misspellings of these terms. Patches with e.g. lidocaine for local anesthetic use were excluded.

### Classification

During ME classification substance, drug brand name, harm, reactions, and error type have been determined. Serious harm was classified in accordance with the WHO definition of a serious adverse event or reaction, i.e. a reaction that results in death, requires inpatient hospitalization or prolongation if existing hospitalization, results in persistent disability/incapacity, or is life threatening [13]. The MedDRA (Medical Dictionary for Regulatory Activities) terminology was used describe the type of adverse reactions reported [14]. In some reports more than one incident was described. In this case incidents considered to causally relate to the first one in the medication process was not included. Also sequentially repeated incidents of the same type in a single report were classified as a single incident. 60 proposed error types, as previously described [15] were used to categorize the MEs (Table 1), based on the free text description of the case. Reports were anonymized and were not traceable back to medical records. Therefore no thorough root cause analysis was possible to perform. Due to lack of information in some incident reports the error type “unclassifiable” was added, and to further analyze our results sub-error types were created for “wrong time” and “wrong dose”. The authors collectively developed the inclusion and exclusion criteria and data extraction. Inclusion of and extraction of data was performed by MH and HL by consensus.

**Table 1:** **Categories of error types used in this study**[15]

**Prescribing**		**Dispensing**	**Administration and monitoring**
**Decision making**	**Communication**		
- Allergy	- Allergy information	- Ambiguous information on label	- Contamination
			- Incompatibility errors
- Calculation error	- Decimal place error	- Incompatibility errors	- Extra dose
- Interaction drug and disease	- Ambiguous drug name	- Contamination	- Lack of control of patient identity
		- Expired drug	
- Interaction between drug and laboratory test	- Ambiguous drug prescription	- Omission of dose	- Omission of dose
- Drug to drug interaction	- P.r.n. prescription without a maximum limit	- Omission of documentation of drug dispensing	- Lack of documentation of the drug administration
- Extra drug	- P.r.n. prescription without a minimum dose interval	- Omission of control of the drug prescription	- Lack of control of agreement between administered drug and prescribed drug
- Omission of a drug prescription			
- Wrong concentration	- Omission of indication for treatment including p.r.n.prescriptions	- Substitution error	- Unordered drug
- Wrong drug form		- Unordered drug	- Wrong dose
- Wrong dose		- Unordered electrolyte	- Wrong patient
- Wrong dosing interval		- Wrong concentration	- Wrong dosing interval
- Wrong drug			
	- Illegible handwriting	- Wrong drug form	- Wrong rate
- Wrong route of administration	- Omission of rate of infusion	- Wrong dose	- Wrong route of administration
- Wrong duration of treatment	- Discrepancy between dose intervals	- Extra dose	- Wrong technique
- Wrong strength/unit		- Wrong strength per unit	- Wrong time
- Omission of ordering laboratory tests	- Discrepancy between indication of dose	- Wrong dilution fluid	- Omission of documentation of side-effects of the drug treatment
	- Wrong transcription		

The error types are listed from left to right in the order of the medication process*.*

## Results

**Figure 1:**
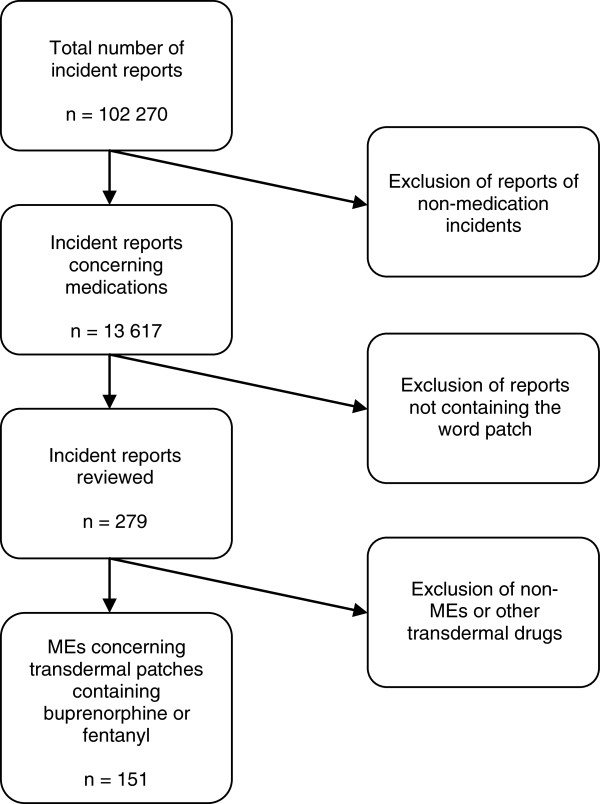
Scheme of case selection.

During the study period a total of 102 270 incident reports were submitted. Selection of opioid patch related MEs are shown in Figure 1. Of all incident reports 13,3% (n = 13 617) concerned medications. Of these, 279 contained at least one of the key terms and were reviewed. Of the reviewed incident reports 149 were selected for this study in accordance with the inclusion criteria. In two incident reports more than one ME was found which resulted in two additional MEs and a total of 151 MEs were included in the analysis. Of the MEs, 66% (n = 100) concerned fentanyl, 28% (n = 42) unknown substance, and 6% (n = 9) buprenorphine. Of the included MEs 48% (n = 73) were reported during 2010 and 2011.

In total, 11 different types of MEs were identified. Some error types may however be found in several stages of the medication process while others are stage specific (Table 1). Administration errors were most commonly reported, and the most frequently occurring error in this category was administration at the wrong time (n = 67)*.* In the prescribing stage wrong transcription errors were most common (n = 9), and in the dispensing stage wrong dose (n = 4), shown in Table 2*.*

**Table 2:** Error types found in the different stages of the medication process

**Error type**	**Prescribing**	**Dispensing**	**Administration**	**Unknown**^**†**^	**Total (%)**
Wrong time	0	1	66		67(44)
Wrong dose	4	4	25	1	34(23)
Omission of dose	0	2	18		20(13)
Wrong transcription	9	0	0		9(6)
Unclassifiable*	0	0	0	6	6(4)
Omission of a drug prescription	5	0	0		5(3)
Lack of documentation of the drug administration	0	0	2		2(1)
Wrong dosing interval	0	0	2		2(1)
Wrong technique	0	0	3		3(2)
Ambiguous drug prescription	1	0	0		1(1)
Lack of control of agreement between administered drug and prescribed drug	0	0	1		1(1)
Wrong route of administration	0	0	1		1(1)
SUM (%)	19(112)	7(5)	8(78)	7(5)	151 (100)

*MEs with no suitable error type^†^*Data didn’t provide enough information to determine in what stage in the medication process the ME occurred.*

MEs in the prescribing stage were often due to incorrect use of electronic systems for prescribing or electronic medical records. In the dispensing stage slips and lapses occurred as causes, and in the administration step lack of compliance with routines, e.g. scheduling visits to outpatients to change patch, was a common cause. The two most frequent error types reported, wrong time and wrong dose, were further analyzed by the addition of subtypes. Wrong time MEs were divided into late (92%, n = 62), early (3% n = 2) and unknown (5%, n = 3). As evident from the short descriptions in the reports scheduling of visits and assigning personnel to the task of changing patches were the most common cause of late administration.

Of the 34 MEs with wrong dose, 13 were cases of too high dose, 10 were cases of too low dose, and 4 with unspecified wrong dose. Of the cases with too high dose, 7 were cases where the old patch were not removed before a new patch was applied.

Harm was reported for 17% (n = 26) of the MEs as shown in Table 3. In two cases there were reports of serious harm. In the first case a patient who ingested a fentanyl patch is described. This patient experienced a respiratory depression and during intubation for respiratory care the patch was discovered in the pharynx. The second case concerns a patient who received a higher dose than prescribed. At admittance to hospital health care personnel found two sets of patches of the prescribed dose on the patient, who was experiencing nausea, vomiting, and pain. The observed adverse reactions (in some cases more than one reaction per ME) were pain (n = 16), withdrawal syndrome (n = 4), fatigue (n = 3), anxiety (n = 2), discomfort (n = 1), dizziness (n = 1), respiratory depression (n = 1), nausea/vomiting (n = 1), tremor (n = 1) and confusional state (n = 1).

**Table 3:** Patient harm by error type

**Error type**	**No harm**	**Harm**	
		**Nonserious**	**Serious**
Omission of dose: n = 20	12	8	0
Wrong time: n = 67	59	8	0
Wrong dose: n = 34	27	6	1
Wrong transcription: n = 9	8	1	0
Wrong technique: n = 3	2	1	0
Wrong route of administration: n = 1	0	0	1
Remaining error types	17	0	0
SUM: n = 151	125	24	2

## Discussion

This is, to our knowledge, the first systematic study assessing and categorizing medication errors concerning transdermal opioid patches. A considerable proportion, 54%, of the MEs assessed in this study constitutes of wrong time - late and omission of doses. The difference between these two error types was the time of discovery; incidents discovered before the next administration were categorized as wrong time – late and incidents discovered at the next scheduled administration or later were categorized as omission of dose. These errors often appeared to be caused by unsatisfactory planning and inadequate patch changing routines. As for all error types, we were unable to perform a full root cause analysis to fully explain what occurred prior to the errors of late administration. It is also important to note that incidents and errors are often consequences of failures in the system rather than solely the act of the individual [16], information that was not available in this dataset on a case by case basis.

Wrong dose constituted 23% of the reported MEs, a diverse error type that was further divided into several subtypes. Analyzing wrong dose – high as well as wrong dose – low, we found that difficulties in handling doses consisting of more than one patch, often with different strengths, sometimes caused problems. Studies have shown that the amount of drug present in used fentanyl patches may be high, in some cases up to 60% of the initial amount [17,18]. As well as potential risk of causing an overdose this could enhance other risks associated with fentanyl use, such as the risk for ADRs. Due to these facts we have considered forgetting to remove old patches when applying a new ones as potentially harmful and this is an important dosage form specific ME. Constituting 21% of the reports of wrong doses, the risks with forgetting old patches on patients may not be considered negligible. Educating healthcare personnel in transdermal opioid pain management and the importance of carefully checking patient for old patches before applying new ones would likely reduce the risk of overdosing.

Of all prescribing MEs, 74% were categorized as wrong transcription or omission of a drug prescription. Mutual for these two error types is that there seemed to be issues concerning electronic prescribing systems and knowledge about how to use it properly. There were also cases where a prescription was noted in the medical record but not transcribed into a separate system for ordering multi-dose packaging. Knowledge about routines related to documenting drug related information in the journal appeared to be another issue. At the same time it is important to note that these systems are likely to reduce mediation errors relating to prescriptions [19,20].

In our study we also found one case of ingestion of a fentanyl patch, which ended up causing the patient serious harm. A multicenter case series concerning whole fentanyl patch ingestion reports hospital admittance in about 78% of the cases with serious adverse reactions like coma, respiratory depression and tachycardia [5]. Patient education regarding the serious risks that this type of misuse can cause could possibly increase the safety in using these types of drugs, with the exception of intentional misuse. The second case of serious harm in our study was categorized as wrong dose – high; a patient who was treated with a relatively high dose, 250 μg, was discovered to have two sets of patches of the prescribed dose on his body at the same time. This most likely contributed to his symptoms that caused admission. Both accidental and voluntary misuse of fentanyl patches causing fatal and nonfatal intoxications are described in the literature [17,21].

The third most commonly reported error type, omission of dose, had the highest proportion of harm (40%). Most common reaction in this category was, as expected, that the patients suffered from pain breakthrough and withdrawal symptoms. Omission of dose, as well as late doses, was often related to inadequate routines for scheduling and performing change of patch.

Interestingly several studies states that using transdermal patches may increase compliance [22,23], but an American study reviewing 644 medication errors with opioids found a higher rate of omission errors with fentanyl patches then with other opioids in different dosage forms, 36% and 12% respectively [1,23,24]. This is in line with our data where reports on omission of doses were relatively common.

The three most commonly reported error types i.e. wrong time, wrong dose, and omission of dose, constitute 80% of all MEs included in this study. Of these 90% occurred in the administration phase of the medication process. We suspected that this uneven distribution of MEs in the medication process partly could be explained by which profession that had predominantly reported the cases included in this study. However this data was not accessible for the years 2004 and 2005, but for the remaining years nurses were submitting the vast majority of the reports (not shown). This may have influenced in what phase of the medication process and what type of errors that were reported. We also suspect that we have an underestimation of MEs in the dispensing stage since neither hospital nor community pharmacies were reporting MEs to the database used in this study.

There are some limitations of this study that needs to be taken into consideration when interpreting the results. Even though reporting incidents to the database used in this study is mandatory for the employees in the county council, underreporting is substantial. It has been estimated that at most 10% of all incidents in our region are reported (personal communication patient safety officer). It is also likely that certain categories of healthcare personnel are more prone to report particular error types, making it challenging to draw conclusion of how common these errors are in the clinical setting. However, the lack of published data on specific problems relating to transdermally delivered drugs warrants this kind of study to capture signals and patterns on MEs with these drugs. In the analysis of MEs we did not have access to medical records therefore some clinical information about the incident, the measures taken and the outcome for the patient was not available. We were not able to check medical record or interview personnel and patients. Doing so would have yielded a deeper understanding of the causes of the errors performed. However, that was neither possible using this data source, nor the scope of this study. Another limitation was the fact that the time of discovery could not always be assessed. Incidents where omission of dose could not be accurately determined were categorized as wrong time-late. As most of the patients had healthcare personnel administering the fentanyl or buprenorphine patches, we only came across very few cases where the patients were self-administrating, a setting with a potentially different pattern of MEs. Furthermore, it is essential to know that ME is a term that is quite imprecisely used in the literature and there are numerous definitions [15,25]. This makes studies in this field difficult to compare. Inclusion of cases considered a ME according to our definition, is to some extent subjective. To reduce the risk of misclassification, only cases where the assessors reached a consensus, were included.

## Conclusions

Using an incident reporting system we were able to describe and characterize medication errors related to transdermal fentanyl and buprenorphine. The vast majority of the reported errors occurred during administration. Improved routines to ascertain correct and timely administration of these drugs and educational interventions focusing on this stage of the medication process are suggested.

## Competing interests

The authors declare that they have no competing interests.

## Authors’ contributions

HL and MH carried out the data retrieval and analysis and drafted the manuscript. SH and HL participated in the design of the study and interpretation of the data. All authors read and approved the final manuscript.

## Pre-publication history

The pre-publication history for this paper can be accessed here:

http://www.biomedcentral.com/2050-6511/15/31/prepub
